# Benzo[*b*]thiophenesulphonamide 1,1-dioxide derivatives inhibit tNOX activity in a redox state-dependent manner

**DOI:** 10.1038/sj.bjc.6602383

**Published:** 2005-02-01

**Authors:** I Encío, D J Morré, R Villar, M J Gil, V Martínez-Merino

**Affiliations:** 1 Department of Health Sciences, Universidad Pública de Navarra, Avda. Barañain, 31008 Pamplona, Spain; 2 Department of Medicinal Chemistry and Molecular Pharmacology, Purdue University, West Lafayette, IN 47907, USA; 3 Department of Applied Chemistry, Universidad Pública de Navarra, Campus Arrosadía, 31006 Pamplona, Spain

**Keywords:** benzo[*b*]thiophenesulphonamides, antineoplasic drugs, ECTO-NOX, tumour-associated NADH oxidase, redox state

## Abstract

Benzo[*b*]thiophenesulphonamide 1,1-dioxide (BTS) derivatives are strong cytotoxic agents that induce reactive oxygen species (ROS) overproduction and apoptosis in tumour cells. Although the precise origin of BTS-induced ROS is not known, a clear correlation between their cytotoxic effect and ability to inhibit a tumour-associated NADH oxidase (tNOX) activity of the plasma membrane has been described. To analyse the putative implication of tNOX in BTS-induced ROS generation, in this work we have synthesised and tested a new BTS derivative, the 6-[*N*-(2-phenylethyl)]benzo[*b*]thiophenesulphonamide 1,1-dioxide. According to its high lipophilicity, this compound showed a strong cytotoxic activity against a panel of six human tumour cell lines, including two human leukaemia (K-562 and CCRF-CEM) and four human solid tumours (HT-29, HTB54, HeLa and MEL-AC). We also tested the ability of this compound to inhibit the tNOX activity and we found an absolute dependence of this inhibition on the redox state of the tNOX: while under reducing conditions, that is, 100 mM GSH, the drug inhibits strongly the NOX activity with an EC_50_ of about 0.1 nM, under oxidising conditions, there is no effect of the drug or just a slight stimulation of activity.

Benzo[*b*]thiophenesulphonamide 1,1-dioxide (BTS) derivatives have been reported as a new class of potential antineoplastic agents (Martínez-Merino *et al*, 2000). These compounds, which were synthesised and analysed in *in vitro* antitumour assays on the basis of theoretic structure–activity COMFA studies of sulphonylureas (Martínez-Merino *et al*, 1995; Gil *et al*, 1999), induce in human leukaemia CCRF-CEM cells a typical process of apoptosis that includes cell shrinkage, phosphatidylserine translocation to the cell surface, mitochondrial dysfunction, caspase activation, chromatin condensation and internucleosomal DNA degradation (Alonso *et al*, 2003). CCRF-CEM cells start to accumulate reactive oxygen species (ROS) within minutes after BTS addition, and these molecules are essential mediators required for the induction of cytochrome 3 mitochondrial release, caspase-3 activation and cell death, as revealed by the fact that these events are prevented by treatment with *N*-acetyl-cysteine (Alonso *et al*, 2003). Although the precise origin of BTS-induced ROS is not known, a clear correlation between their cytotoxic effect and ability to inhibit an NADH oxidase (NOX) activity found in the plasma membrane and conditioned culture medium of CCRF-CEM cells has been described (Alonso *et al*, 2001).

ECTO-NOX are cell surface-associated and growth-related protein oxidases that exhibit two different activities, protein disulphide-thiol interchange and hydroquinone (or NADH) oxidation, that alternate to yield oscillatory patterns (Morré and Morré, 2003). Two forms of ECTO-NOX have been detected in sera of cancer patients (Wang *et al*, 2003): a widely distributed constitutive NOX (CNOX) of the mammalian cell surface that has a period length of 24 min (Sedlak *et al*, 2001) and is resistant to inhibition by quinone site inhibitors (Chueh *et al*, 2002a) like the vainilloid capsaicin or the antitumour sulphonylurea *N*-4-(methylphenylsulphonyl)-*N*′-(4-chlorophenyl)urea (LY181984), and a tumour-associated NOX (tNOX) with a period length of 22 min (Chueh *et al*, 2002a) that is inhibited by capsaicin (Morré *et al*, 1997a) or LY181984 (Morré and Reust, 1997b) and is low or absent from sera of individuals not diagnosed as having cancer (Morré *et al*, 1997a). The tNOX protein is also specifically inhibited in HeLa and human mammary adenocarcinoma cells by (−)-epigallocatechin-3-gallate (EGCg) (Morré *et al*, 2000), the principal catechin of green tea; EGCg also inhibited growth of transformed cells in culture. Since this action appears to result from an effect on regulation of cell cycle progression and induction of apoptosis (Ahmad *et al*, 1997, 2000, 2002; Gupta *et al*, 2000) rather than from an unspecific antioxidant function (Salucci *et al*, 2002), tNOX protein has been proposed as the molecular target on cancer cells to explain their specific inhibition of growth by EGCg (Morré *et al*, 2000).

The putative implication of tNOX in BTS-induced ROS generation and the fact that some enzymes related with ROS control such as the glutathione reductase and the glutathione *S*-transferase present a hydrophobic pocket near their active site (Karplus and Schulz, 1989; Chern *et al*, 2000) led us to synthesise and analyse new derivatives of the benzo[*b*]thiophene 1,1-dioxide carrying hydrophobic substituents of different length and grade of flexibility on the sulphonamide group and, in some cases, a clear correlation between the lipophilicity (log *P*) and the cytotoxic effect of these compounds was observed (Villar *et al*, 2004). Here we describe the synthesis and cytotoxic activity of 6-[*N*-(2-phenylethyl)]benzo[*b*]thiophenesulphonamide 1,1-dioxide (BTS-2), a new BTS derivative with increased flexibility, high lipophilicity (log *P*=2.82) and a predicted low toxicity for its putative metabolites, and we show its ability to specifically inhibit the tNOX activity and the absolute dependence of this inhibition on the redox state of the tNOX.

## MATERIALS AND METHODS

### Chemistry

Benzo[*b*]thiophenesulphonamide 1,1-dioxide (BTS-1) was prepared as previously described (Martínez-Merino *et al*, 2000).

**Scheme 1:**
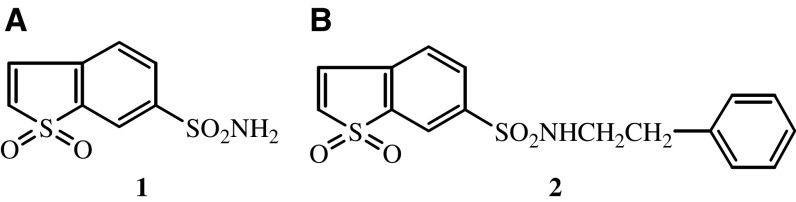
i: Acetic acid, H_2_O_2_ 30% (v v^−1^), reflux, 30 min; ii: nitric acid 100%; iii: Fe/CINH_4_, ethyl alcohol/water 50%; iv: NaNO_2_, HCI (ac); SO_2_/CuCl, acetic acid; v: CH_2_Cl_2_; triethylamine.

The synthesis of BTS-2 was carried out by the usual methods described for the synthesis of sulphonamide derivatives (Villar *et al*, 2004), that is, through the treatment of the sulphonyl chloride derivative with ammonia or amines (Scheme 1). The chlorosulphonyl derivative was obtained from the 6-aminobenzo[*b*]thiophene 1,1-dioxide by the Meerwein's method (Meerwein *et al*, 1957) (treatment of diazonium salts with sulphonyl chloride in the presence of cuprous chloride), and then treated with phenetylamine to give the BTS-2 (28.1% yield). The previous amine derivative was produced by reduction of 6-nitrobenzo[*b*]thiophene 1,1-dioxide, and the last one was synthesised according to procedures previously published (Challenger and Clapham, 1948) (60% yield). The oxidation of benzo[*b*]thiophene was carried out with 30% hydrogen peroxide.

### Cell culture

American Type Culture Collection (ATCC, Manassas, VA) or European Collection of Cell Cultures (ECACC, Porton Down, Salisbury, UK) provided human tumour cell lines. Six cell lines were used: two human leukaemia (K-562 and CCRF-CEM) and four human solid tumours, one colon carcinoma (HT-29), one lung carcinoma (HTB54), one cervix epitheloid carcinoma (HeLa) and one melanoma (MEL-AC). MEL-AC cells were kindly provided by Dr Natalia López-Moratalla (Universidad de Navarra, Pamplona, Spain). Human lung fibroblasts (HLFs) were kindly provided by Dr Markus Nabholzs (ISREC, Epalinges, Switzerland). Cells were grown in RPMI 1640 medium (Life Technologies, Barcelona, Spain) supplemented with 10% fetal calf serum, 2 mM L-glutamine, 100 U ml^−1^ penicillin, 100 *μ*g ml^−1^ streptomycin and 10 mM HEPES buffer (pH 7.4).

### Cytotoxicity assay

The cytotoxic effect of each substance was tested at five different doses between 0.01 and 100 *μ*M. Each substance was initially dissolved in DMSO at a concentration of 0.1 M, and serial dilutions were prepared using culture medium. The plates with cells from the different lines, to which media containing the substance under test were added, were incubated for 72 h at 37°C in a humidified atmosphere containing 5% CO_2_. Cytotoxicity was then determined by a colorimetric microassay based on the use of MTT (Mosmann, 1983). Results are expressed as GI_50_, concentration that reduced by 50% the growth of treated cells with respect to untreated controls.

### Protein isolation and determination

Cell membrane NOX activity (Cellex) was released in soluble form from HeLa cells (whole cells) as previously described (Del Castillo-Olivares *et al*, 1998).

Proteins were determined by the bicinchoninic acid/copper assay (Smith *et al*, 1985) obtained from Pierce using bovine serum albumin as standard.

### NADH oxidase activity measurement

NADH oxidase activity was determined as the disappearance of NADH at 340 nm in a reaction mixture containing 25 mM Tris-Mes buffer (pH 7.2), 1 mM KCN to inhibit any potential mitochondrial oxidase activity and 150 *μ*M NADH at 37°C and continuous stirring (Wang *et al*, 2003). Assays were initiated by addition of NADH. Absorbance was recorded for 1 min after adding NADH and a millimolar extinction coefficient of 6.22 was used to determine specific activity.

## RESULTS

**Figure 1:**
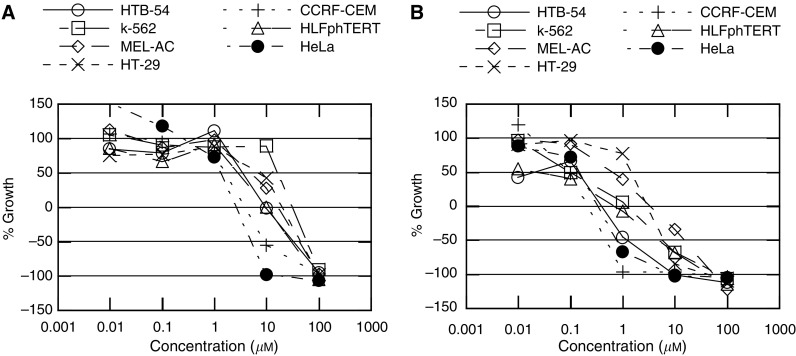
Structure of BTS used in this work.

**Figure 2:**
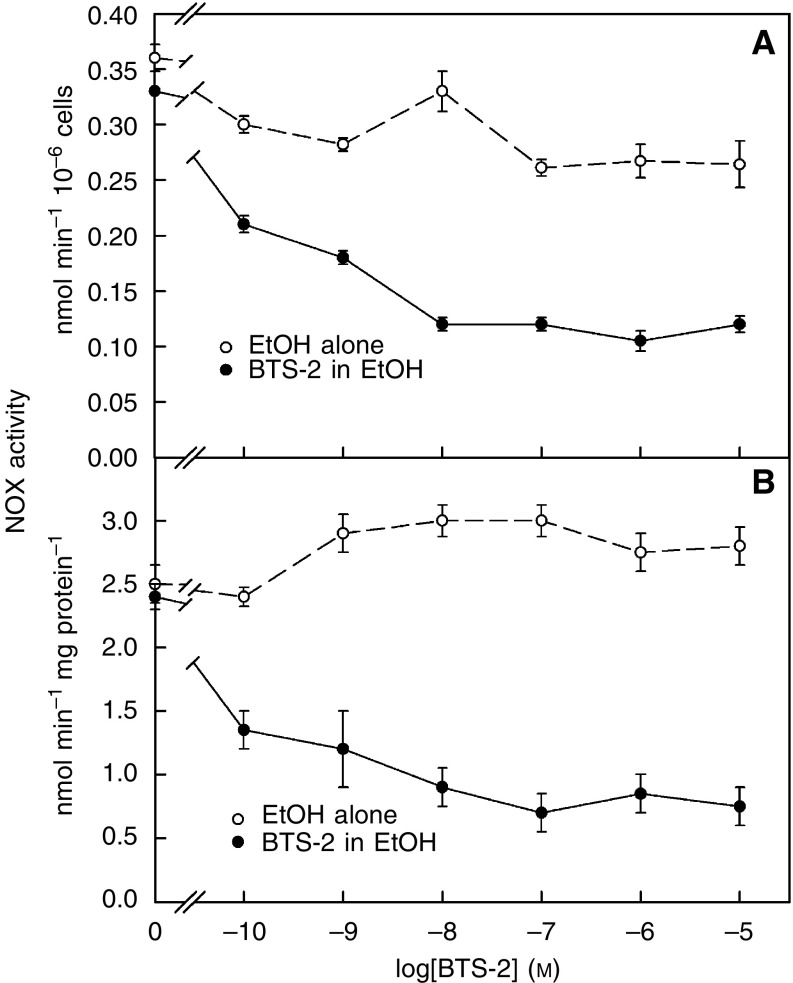
Determination of the cytotoxic effect of (**A**) BTS-1 and (**B**) BTS-2. Cells were incubated in the presence of every compound at the indicated concentration for 72 h. Cytotoxicity was then determined by a colorimetric microassay based on the use of MTT (Mosmann, 1983). Values represent means±s.d. derived from three independent experiments each performed in quadruplicate.

The cytotoxic activity of BTS-1 and BTS-2 (Figure 1) was tested against a panel of human tumour cell lines, including cervix epitheloid carcinoma (HeLa), lymphocytic leukaemia (CCRF-CEM), myelocytic leukaemia (K-562), melanoma (MEL-AC), human colon carcinoma (HT-29) and lung carcinoma (HTB-54), and also against normal HLFs growing in culture. Cells were grown in RPMI 1640 medium (Life Technologies) supplemented with 10% fetal calf serum, 2 mM L-glutamine, 100 u ml^−1^ penicillin, 100 *μ*g ml^−1^ streptomycin and 10 mM HEPES buffer (pH 7.4). The cytotoxic effect of each substance was tested at five different doses between 0.01 and 100 *μ*M. The plates with cells from the different lines, to which media containing the substance under test were added, were incubated for 3 days and cell survival was then determined by an MTT-based colorimetric assay (Mosmann, 1983). Results are expressed as GI_50_, concentration that reduced by 50% the growth of treated cells with respect to untreated controls. As shown in Table 1, growth of every cell line was clearly affected by the tested compounds, and is worth noting that according to its higher lipophilicity, BTS-2 was more cytotoxic against each one of the tested cell lines than the lead compound (BTS-1). Moreover, activity of BTS-2 was in the same order of magnitude as commercial doxorubicin against K-562 tumour cells and normal lung fibroblasts growing in culture. Figure 2 shows curves with the original data from which the GI_50_ values for BTS-1 and BTS-2 were calculated.

**Figure 3:**
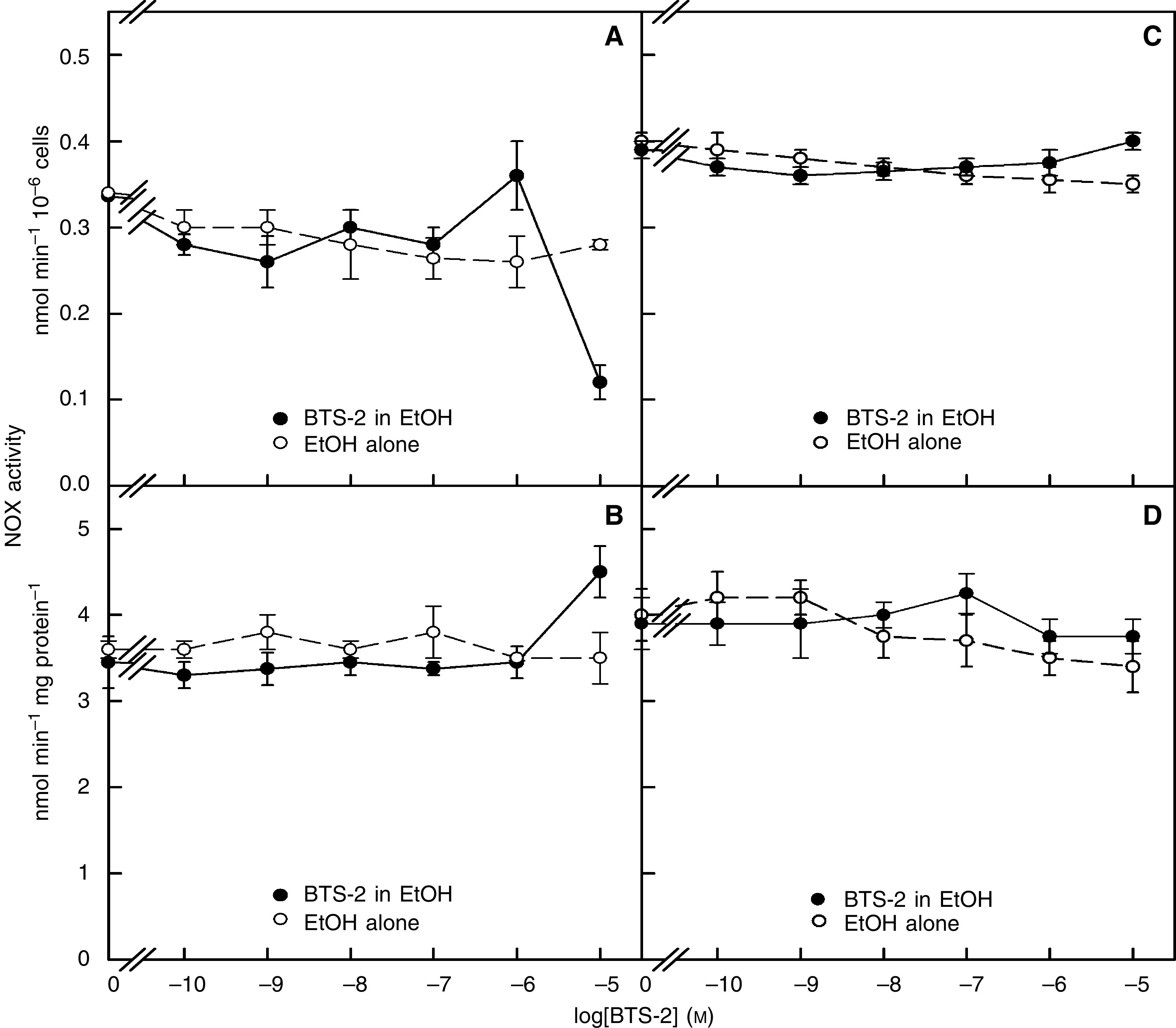
Dose-dependent inhibition by BTS-2 of NOX activity of (**A**) HeLa cells and (**B**) Cellex preparations in the presence of 100 mM GSH (solid symbols). Assay was based on spectrophotometric measurement of NADH disappearance at 340 nm. The compound was dissolved in ethanol and compared to solvent alone (open symbols).

It has been previously shown that BTS-1 and some related diarylsulphonylureas (DSU) are able to inhibit a tumour cell-specific NOX activity present in the membrane of CCRF-CEM cells (Alonso *et al*, 2001). As we had said before, until now tNOX activity is the only cell target proposed to explain the specific inhibition of growth of cancer cells induced by the antitumour sulphonylurea LY181984 (Morré *et al*, 1995a, 1995b) and the green tea catechin EGCg (Morré *et al*, 2000). Since BTS-2 was designed on the basis of experimental results obtained with BTS-1 and other BTS that are structurally related with DSU, we decided to further analyse the action of BTS-2 on tNOX activity. For these purpose, we performed standard NOX assays using either a preparation released at pH 5 from HeLa cells obtained from Cellex Biosciences (Minneapolis, USA), which is a mixture of NOXs (Del Castillo-Olivares *et al*, 1998), or from intact HeLa cells. NADH is an impermeant substrate and, therefore, can be used to measure cell surface NADH activity with whole cells. Results with isolated plasma membrane and with whole cells were similar. NADH oxidase activity was determined as the disappearance of NADH measured at 340 nm. The compound was dissolved in ethanol and compared to solvent alone. As shown in Figure 3, in the presence of reducing conditions, that is, 100 *μ*M GSH, the drug inhibited strongly the NOX activity of both the intact HeLa (Figure 3A) and Cellex (Figure 3B) preparations with an EC_50_ of about 0.1 nM. The figure also shows small stimulations by ethanol (chemical hormesis) that are not unusual for the cell surface NOX activities (Morré, 2000). Although both the Cellex and intact HeLa preparations contain a cancer-specific and drug-inhibited tNOX and a constitutive and drug-resistant NOX, the data show that only tNOX is being inhibited, and indicate that this inhibition, which is dose dependent, is almost complete at 0.1 *μ*M. Otherwise, the residual NOX activity corresponding to CNOX would approach zero at the high drug concentration. In agreement with this, NOX activities of plasma membranes purified from soybean (*Glycine max*), which contain only CNOX, were unaffected by BTS-2 over the concentration range 10^−11^–10^−5^ M (*N*=4) (not shown).

**Figure 4:**
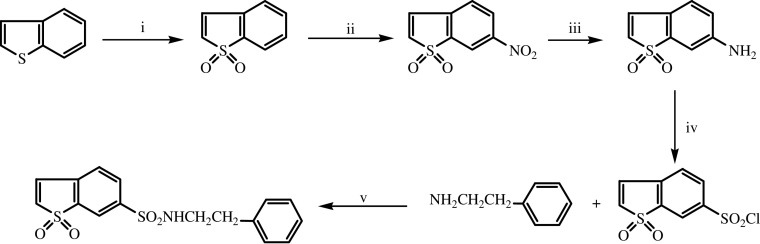
Lack of inhibition of the NOX activity by BTS-2 under oxidising conditions (solid symbols). (**A**) Normal NOX assays performed with intact HeLa cells in which the preparations were first reduced with 100 mM GSH for 10 min and then fully oxidised by addition of 0.03% hydrogen peroxide for 10 min. (**B**) As in (**A**) but with 50 ml of the Cellex preparation. (**C**) As in (**A**) but with assay in the presence of 100 mM GSSG. (**D**) As in (**B**) but with assay in the presence of 100 mM GSSG. The compounds were dissolved in ethanol and compared to solvent alone (open symbols). The ethanol concentration was 0.16% maximum.

The effect of the drug on the NOX activity under oxidising conditions is analysed in Figure 4. As can be seen, when the oxidising conditions were provided by treatment of HeLa cells with hydrogen peroxide (Figure 4A), the derivative inhibited only at the highest concentration of 10 *μ*M. Moreover, with Cellex preparations (Figure 4B), stimulation was recorded at 10 *μ*M. As with hydrogen peroxide, when the assays were performed in the presence of 100 *μ*M GSSG (Figure 4C and D), we could see no inhibition of the NOX activity, only a slight stimulation. Thus, it can be concluded that while under reducing conditions BTS-2 strongly inhibits tNOX, under oxidising conditions there is no effect of the drug or just a slight stimulation of activity. The absolute dependency of inhibition on redox state of tNOX has also been described with the antitumour sulphonylureas (Morré *et al*, 1998).

## DISCUSSION

We have previously shown that BTS derivatives are strong cytotoxic agents that induce ROS overproduction and apoptosis in tumour cells (Alonso *et al*, 2003). We have also shown that while the BTS derivatives that exhibit this cytotoxic activity are able to inhibit a NOX activity of the plasma membrane of leukaemia CCRF-CEM cells, those that lack cytotoxic activity also lack this inhibitory effect (Alonso *et al*, 2001), thus suggesting that ECTO-NOX proteins could provide a molecular target for the induction of ROS production by BTS derivatives. Here we report that BTS-2, a new BTS derivative with high lipophilicity and improved cytotoxic activity, specifically inhibits the tNOX activity of HeLa cells, and we show that this inhibition is dependent on the redox state of tNOX.

A number of motifs within tNOX, including an NADH binding site, a quinone binding site and sites for protein disulphide-thiol interchange, account for the range of its functional activities (Chueh *et al*, 2002b). The drug response of tNOX has been studied extensively with LY181984 (Morré *et al*, 1998). Activity is inhibited or stimulated by this sulphonylurea depending on the redox environment of the protein. Ubiquinone competes with both LY181984 binding and inhibition of enzymatic activities and so the drug, like capsaicin (Morré *et al*, 1995c), EGCg (Morré *et al*, 2000) and adriamycin (Morré *et al*, 1997c), which also inhibit activity, is considered to occupy the quinone binding site. Interestingly, while the normal CNOX isoform and tNOX share functional characteristics, CNOX appears to lack the quinone binding motif EEMTE (Chueh *et al*, 2002a), a fact that could explain why both enzymes differ primarily in their sensitivity to these drugs. Since we show here that under reducing conditions BTS-2 inhibits strongly the NOX activity of both intact HeLa cells and Cellex preparations containing both tNOX and CNOX, while ECTO-NOX activities of plasma membranes from soybean, which contain only CNOX, are unaffected by this drug, the possibility that BTS derivatives exert their specific inhibitory action on tNOX activity through binding to the quinone site should be considered.

It has been described that the tNOX activity can be inhibited by thiol reagents such as the *N*-ethyl-maleimide (NEM) (Morré *et al*, 1995a, 1995b). It has also been reported that compounds carrying the benzo[*b*]thiophene 1,1-dioxide nucleus, like BTS-1 and -2, are thiol reagents (Bordwell *et al*, 1954). For this reason, it is very interesting that the C2–C3 dihydro derivative of BTS-1, which was reported to be inactive both in the cytotoxicity and enzymatic assays (Alonso *et al*, 2001), cannot be considered a thiol reagent. Since this compound and BTS-1 have identical molecular sizes and capacities to interact through polar groups, hydrogen bonds and/or hydrophobic interactions with putative receptor sites, activity of BTS-1 can be attributed to the C2–C3 double bond reactivity. In agreement with this, the C3-methyl derivative of BTS-1 shows no cytotoxic activity (Villar *et al*, 2004), a result that can be attributed to its impaired reactivity towards thiol groups. Thus, the possibility that BTS derivatives exert their inhibitory action on tNOX activity by interacting with cysteines of the active sites for protein disulphide-thiol interchange should also be considered. The fact that BTS actions over cell growth are prevented by previous treatment of the cells with *N*-acetyl-cysteine (Alonso *et al*, 2003) and our finding that under oxidising conditions there is no effect of BTS-2 on the tNOX activity also support this idea.

Interference with DNA and RNA is generally considered to account for the cytotoxicity of anthracycline antitumour drugs. However, it has been shown that doxorubicin is cytotoxic even without entering the cells (Tritton and Yee, 1982), and inhibition of tNOX activity by doxorubicin (EC_50_=0.7 nM) has been described (Morré *et al*, 1997c; Hedges *et al*, 2003). Interestingly, when cell growth is inhibited, redox activities of the plasma membrane are also inhibited, and thus NOX activity of the plasma membrane has been suggested as a growth-related doxorubicin target at the surface of cancer cells (Kim *et al*, 2002; Hedges *et al*, 2003). For this reason, the fact that BTS-2 acts in the same order of magnitude as doxorubicin against K-562 tumour cells and also against normal HLFs that should have a low or even absent tNOX activity rises the possibility of additional sites of action for this drug. Further experiments are required to clarify this fact.

## Figures and Tables

**Table 1:** Cytotoxic activities (GI_50_, *μ*M inhibition of cell growth) against tumor cell lines and lipophilicity

**Compound**	**HeLa**	**CCRF-CEM**	**K-562**	**MEL-AC**	**HT-29**	**HTB-54**	**HLF**	**log *P***
BTS-1	2.2	2.86	29.41	7.14	8.55	5.89	5.04	−0.03
BTS-2	0.24	0.1	0.07	0.81	2.52	0.23	0.04	2.82
Dx	0.02	0.03	0.02	0.01	ND	ND	0.01	ND

Dx: doxorubicin; ND: not determined.

## References

[bib1] Ahmad N, Adhami VM, Gupta S, Cheng P, Mukhtar H (2002) Role of the retinoblastoma (pRb)-E2F/DP pathway in cancer chemopreventive effects of green tea polyphenol epigallocatechin-3-gallate. Arch Biochem Biophys 398: 125–131 11811957 10.1006/abbi.2001.2704

[bib2] Ahmad N, Feyes DK, Nieminen AL, Agarwal R, Mukhtar H (1997) Green tea constituent epigallocatechin-3-gallate and induction of apoptosis and cell cycle arrest in human carcinoma cells. J Natl Cancer Inst 89: 1881–1886 9414176 10.1093/jnci/89.24.1881

[bib3] Ahmad N, Gupta S, Mukhtar H (2000) Green tea polyphenol epigallocatechin-3-gallate differentially modulates nuclear factor kappaB in cancer cells *versus* normal cells. Arch Biochem Biophys 376: 338–346 10775421 10.1006/abbi.2000.1742

[bib4] Alonso MM, Asumendi A, Villar J, Gil MJ, Martínez-Merino V, Encío I, Migliaccio M (2003) New benzo(*b*)thiophenesulphonamide 1,1-dioxide derivatives induce a reactive oxygen species-mediated process of apoptosis in tumor cells. Oncogene 22: 3759–3769 12802283 10.1038/sj.onc.1206435

[bib5] Alonso MM, Encío I, Martínez-Merino V, Gil MJ, Migliaccio M (2001) New cytotoxic benzo(*b*)thiophenilsulfonamide 1,1-dioxide derivatives inhibit a NADH oxidase located in plasma membranes of tumor cells. Br J Cancer 85: 1400–1402 11720481 10.1054/bjoc.2001.2083 PMC2375244

[bib6] Bordwell FG, Chapman RD, McKellin WH (1954) Benzothiophen chemistry. VI. A peroxide effect in the addition of thiophenols to benzothiophene 1-dioxide. J Am Chem Soc 76: 3637–3639

[bib7] Challenger F, Clapham PH (1948) Some derivatives of thionaphthen. J Chem Soc (oct): 1615–1618 18101467 10.1039/jr9480001615

[bib8] Chern MK, Wu TC, Hsieh CH, Chou CC, Liu LF, Kuan IC, Yeh YH, Hsiao CD, Ming F, Tam MF (2000) Tyr115, gln165 and trp209 contribute to the 1, 2-epoxy-3-(*p*-nitrophenoxy)propane-conjugating activity of glutathione *S*-transferase cGSTM1-1. J Mol Biol 300: 1257–1269 10903867 10.1006/jmbi.2000.3904

[bib9] Chueh PJ, Kim C, Cho N, Morré DM, Morré DJ (2002a) Molecular cloning and characterization of a tumor-associated, growth-related, and time-keeping hydroquinone (NADH) oxidase (tNOX) of the HeLa cell surface. Biochemistry 41: 3732–3741 11888291 10.1021/bi012041t

[bib10] Chueh PJ, Morré DM, Morré DJ (2002b) A site-directed mutagenesis analysis of tNOX functional domains. Biochim Biophys Acta 1594: 74–83 11825610 10.1016/s0167-4838(01)00286-2

[bib11] Del Castillo-Olivares A, Yantiri F, Chueh PJ, Wang S, Sweeting M, Sedlak D, Morré DM, Burgess J, Morré DJ (1998) A drug-responsive and protease-resistant peripheral NADH oxidase complex from the surface of HeLa S cells. Arch Biochem Biophys 358: 125–140 9750173 10.1006/abbi.1998.0823

[bib12] Gil MJ, Mañú M, Arteaga C, Migliaccio M, Encío IJ, González A, Martínez-Merino V (1999) Synthesis and cytotoxic activity of *N*-(2-pyridylsulfenyl)urea derivatives. A new class of potential antineoplastic agents. Bioorg Med Chem Lett 9: 2321–2324 10476861 10.1016/s0960-894x(99)00373-x

[bib13] Gupta S, Ahmad N, Nieminen AL, Mukhtar H (2000) Growth inhibition, cell-cycle dysregulation, and induction of apoptosis by green tea constituent (-)-epigallocatechin-3-gallate in androgen-sensitive and androgen-insensitive human prostate carcinoma cells. Toxicol Appl Pharmacol 164: 82–90 10739747 10.1006/taap.1999.8885

[bib14] Hedges KL, Morré DM, Wub LY, Morré DJ (2003) Adriamycin tolerance in human mesothelioma lines and cell surface NADH oxidase. Life Sci 73: 1189–1198 12818726 10.1016/s0024-3205(03)00421-1

[bib15] Karplus PA, Schulz GE (1989) Substrate binding and catalysis by glutathione reductase as derived from refined enzyme: substrate crystal structures at 2 Å resolution. J Mol Biol 210: 163–180 2585516 10.1016/0022-2836(89)90298-2

[bib16] Kim C, Crane FL, Faulk WP, Morré DJ (2002) Purification and characterization of a doxorubicin-inhibited NADH-quinone (NADH-ferricyanide) reductase from rat liver plasma membranes. J Biol Chem 277: 16441–16447 11875069 10.1074/jbc.M112311200

[bib17] Martínez-Merino V, Gil MJ, Encío I, Migliaccio M, Arteaga C (2000) Benzo[*b*]thiophene sulfonamide-1,1-dioxido derivatives and their use as antineoplastic agents. WO Patent 00/63202

[bib18] Martínez-Merino V, Martínez-González A, González A, Gil MJ (1995) 3D-QSAR of the diarylsulphonylureas as antineoplastic agents. In QSAR and Molecular Modelling: Concepts, Computational Tools and Biological Applications Sanz F, Giraldo J, Manaut F (eds) pp 478–480. Barcelona: Prous Science Publishers

[bib19] Meerwein H, Dittmer G, Gollner R (1957) Verfahren zur herstellung aromatischer sulfonsäurechloride, eine neue modifikation der Sandmeyerschen reaktion. Chem Ver 90: 841–1178

[bib20] Morré DJ, Wu LY, Morré DM (1995b) The antitumor sulfonylurea *N*-(4-methylphenylsulfonyl)-*N*′-(4-chlorophenyl) urea (LY181984) inhibits NADH oxidase activity of HeLa plasma membranes. Biochim Biophys Acta 1240: 11–17 7495842 10.1016/0005-2736(95)00164-7

[bib21] Morré DJ (2000) Chemical hormesis in cell growth: a molecular target at the cell surface. J Appl Toxicol 20: 157–163 10715615

[bib22] Morré DJ, Bridge A, Wu LY, Morré DM (2000) Preferential inhibition by (−)-epigallocatechin-3-gallate of the cell surface NADH oxidase and growth of transformed cells in culture. Biochem Pharmacol 60: 937–946 10974202 10.1016/s0006-2952(00)00426-3

[bib23] Morré DJ, Caldwell S, Mayorga A, Wu LY, Morré DM (1997a) NADH oxidase activity from sera altered by capsaicin is widely distributed among cancer patients. Arch Biochem Biophys 342: 224–230 9186482 10.1006/abbi.1997.0110

[bib24] Morré DJ, Chueh PJ, Morré DM (1995c) Capsaicin inhibits preferentially the NADH oxidase and growth of transformed cells in culture. Proc Natl Acad Sci USA 92: 1831–1835 7892186 10.1073/pnas.92.6.1831 PMC42376

[bib25] Morré DJ, Kim C, Paulik M, Morré DM, Faulk WP (1997c) Is the drug-responsive NADH oxidase of the cancer cell plasma membrane a molecular target for adriamycin. J Bioenerg Biomembr 29: 269–280 9298712 10.1023/a:1022414228013

[bib26] Morré DJ, Morré DM (2003) Cell surface NADH oxidases (ECTO-NOX proteins) with roles in cancer, cellular time-keeping, growth, aging and neurodegenerative diseases. Free Radic Res 37: 795–808 14567438 10.1080/1071576031000083107

[bib27] Morré DJ, Morré DM, Stevenson J, Mackellar W, McClure D (1995a) HeLa plasma membranes bind the antitumor sulfonylurea LY181984 with high affinity. Biochim Biophys Acta 1244: 133–140 7766649 10.1016/0304-4165(94)00211-f

[bib28] Morré DJ, Reust T (1997b) A circulating form of NADH oxidase activity responsive to the antitumor sulfonylurea *N*-4-(methylphenylsulfonyl)-*N*′-(4-chlorophenyl)urea (LY181984) specific to sera from cancer patients. J Bioenerg Biomembr 29: 281–289 9298713 10.1023/a:1022466212083

[bib29] Morré DJ, Wu LY, Morré DM (1998) Response of a cell-surface NADH oxidase to the antitumor sulfonylurea *N*-(4-methylphenylsulfonyl)-*N*′-(4-chlorophenylurea) (LY181984) modulated by redox. Biochim Biophys Acta 1369: 185–192 9518604 10.1016/s0005-2736(97)00202-2

[bib30] Mosmann T (1983) Rapid colorimetric assay for cellular growth and survival: application to proliferation and cytotoxicity assays. J Immunol Methods 65: 55–59 6606682 10.1016/0022-1759(83)90303-4

[bib31] Salucci M, Stivala LA, Maiani G, Bugianesi R, Vannini V (2002) Flavonoids uptake and their effect on cell cycle of human colon adenocarcinoma cells (Caco2). Br J Cancer 86: 1645–1651 12085217 10.1038/sj.bjc.6600295 PMC2746583

[bib32] Sedlak D, Morré DM, Morré DJ (2001) A drug-unresponsive and protease-resistant CNOX protein from human sera. Arch Biochem Biophys 386: 106–116 11360993 10.1006/abbi.2000.2180

[bib33] Smith PK, Krohn RI, Hermanson GT, Mallia AK, Gartner FH, Provenzano MD, Fujimoto EK, Goeke NM, Olson BJ, Klenk DC (1985) Measurement of protein using bicinchoninic acid. Anal Biochem 150: 76–85 3843705 10.1016/0003-2697(85)90442-7

[bib34] Tritton RR, Yee G (1982) The anticancer agent adriamycin can be actively cytotoxic without entering cells. Science 217: 248–250 7089561 10.1126/science.7089561

[bib35] Villar R, Encío I, Migliaccio M, Gil MJ, Martínez-Merino V (2004) Synthesis and cytotoxic activity of lipophilic sulphonamide derivatives of the benzo[*b*]thiophene 1,1-dioxide. Bioorg Med Chem 12: 963–968 14980609 10.1016/j.bmc.2003.12.012

[bib36] Wang S, Morré DM, Morré DJ (2003) Sera from cancer patients contain two oscillating ECTO-NOX activities with different period lengths. Cancer Lett 190: 135–141 12565167 10.1016/s0304-3835(02)00616-x

